# Transit time flow measurement in arterial grafts

**DOI:** 10.1186/s13019-024-02670-6

**Published:** 2024-04-16

**Authors:** Dror B. Leviner, John D. Puskas, David P. Taggart

**Affiliations:** 1 Department of Cardiac Surgery, Carmel Medical Center, Haifa, Israel; 2 The Ruth & Baruch Rappaport Faculty of Medicine, Technion-Israel Institute of Technology, Haifa, Israel; 3 Devision of Cardiothoracic Surgery, Emory University School of Medicine, Atlanta, GA USA; 4 Department of Cardiac Surgery, John Radcliffe Hospital, University of Oxford, Oxford, UK

## Abstract

Coronary artery bypass grafting (CABG) is one of the foundations of treatment for coronary artery disease. While it has improved substantially since its inception more than 50 years ago, including a rising use of multiple arterial grafting, intraoperative quality assessment is yet to be disseminated as an integral part of the procedure. Herein we review the fundamentals of intraoperative quality assessment in CABG using transient time flow measurement (TTFM) with a focus on its use in arterial grafting.

## Introduction

Coronary artery bypass grafting (CABG) is the most common procedure in cardiac surgery. While intraoperative quality assessment with echocardiography is an integral part of mitral valve repair, for instance, intraoperative quality assessment has not been widely adopted in CABG with overall rates of use at around 30% but enormous geographical variation [1]. Transient time flow measurement (TTFM) is the most common method of quality assessment in coronary surgery, mainly due to its ease of use. While current guidelines recommend the routine use of TTFM in CABG (class IIa, level of evidence B) [2], there are barriers to widespread adoption, most likely lack of clear cutoff values, knowledge gaps, and the learning curve after adoption of a new technology. Moreover, like any other test in medicine, TTFM has both a false positive (good graft, poor TTFM measurements) and false negative (poor graft, adequate measurements), both at unknown rates [3] Consequently, and as discussed subsequently, an expert consensus panel has recently made recommendations to standardize the use of TTFM [4].

## Role of TTFM in coronary surgery

**Fig. 1:**
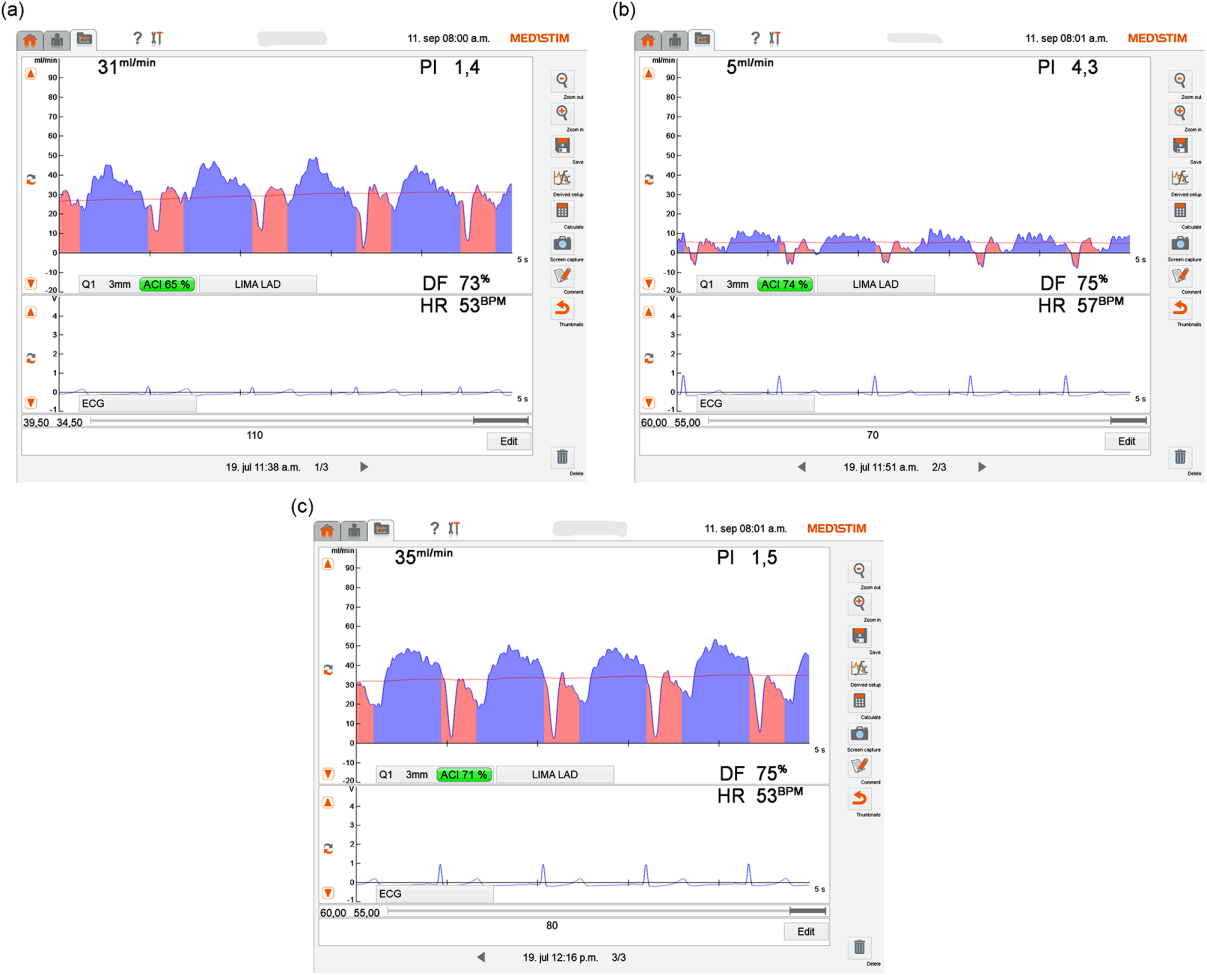
TTFM measurement of a LIMA to LAD graft. Use of the imaging probe showed an intimal flap in the anastomosis (which is most probably the result from a plaque removed when an endarterectomy was preformed). (**a**) The surgeon did not revise the graft initially, as the TTFM parameters were good (pre-protamine). (**b**) However, the TTFM parameters dropped post-protamine and the graft was revised. (**c**) The flap was removed, and the MGF & PI improved

CABG has proven superior to percutaneous coronary intervention (PCI) in several patient groups including those with multivessel disease, those with diabetes, and those with left main disease [5, 6]. To keep improving and maintaining this advantage, multiple aspects of CABG must be addressed, including quality assessment. TTFM, especially when coupled with high-frequency ultrasound (HFUS), has been shown to result in improved outcomes. The Registry for Quality Assessment with Ultrasound Imaging and Transit-Time Flow Measurement in Cardiac Bypass Surgery (REQUEST) study [7] showed that when used by experienced surgeons TTFM assessment is associated with a change in the planned surgical procedure in 25.5% of patients including graft revision in 7.8% of patients (Fig. 1). This was reflected in extremely low post-operative adverse events. In a meta-analysis [8] studying the impact of TTFM adoption on CABG procedures (including 8943 patients and 15,673 grafts) the revision rate was 4.3%. (but, of all grafts classified as abnormal only 25% were revised). Some [9], though not all [10], studies included in the meta-analysis showed a significant reduction in post-operative adverse events (including short term post-operative mortality, post operative myocardial infarction, use of intra-aortic balloon pump, and overall morbidity [8] after TTFM adoption.

## Benefits of arterial grafts

Another technical aspect of coronary surgery that requires improvement is the routine use of multiarterial grafting. While use of the left internal mammary artery (LIMA) to the left anterior descending (LAD) artery is considered essential to routine CABG since the seminal trials published in the 1980’s [11], the use of a second arterial graft (termed multiarterial grafting or MAG) is not yet routine, let alone total arterial grafting (TAG). There is a large body of evidence showing survival benefit with the use of bilateral internal mammary arteries (BIMA) over LIMA alone including mostly observational studies [12] and meta-analysis with a large sample size [13]. Proving the benefit of MAG over LIMA in the setting of a randomized controlled trial (RCT) has proven more challenging [14] which might pose one of the hurdles to a higher adoption rate, given the higher technical complexity of MAG and the fear of sternal wound infection. The patency of the right internal mammary artery (RIMA) is more dependent on the coronary target then on the configuration in which the RIMA is used [15] Data also shows the benefits of using the radial artery as a second arterial conduit in reducing long term adverse events [16].

## Flow parameters in TTFM

Before discussing the use of TTFM in arterial grafts, we will briefly review the different parameters used in TTFM (All flow parameters in this review refer to the MiraQ system, Medistim, Oslo, Norway). There are four variables derived from TTFM measurements in CABG: 1) Mean graft flow (MGF), Pulsatility index (PI), 3) Diastolic filling (DF), and 4) backward flow (BF). MGF is reported in mL per minute and is influenced by multiple factors, primarily the graft size and quality, target vessel size and the severity of stenosis. MGF is considered abnormal below 15–20 mL/min. This has been correlated with both short and mid-term patency of grafts, including the IMA [17]. The PI is an absolute value calculated by dividing the difference between the peak flow and the minimum flow by the MGF calculated across 5 cardiac cycles. The PI represents the resistance to flow and is influenced by all types of resistance, including anastomotic quality, graft stenosis, and native coronary artery stenosis distal to the anastomosis. A PI > 5 is considered abnormal. DF is the percentage of diastolic graft flow. All grafts show a diastolic flow pattern which is higher in coronary arteries supplying the left ventricle. DF is higher in the distal portion of the graft. A DF of < 50% is considered abnormal. BF is the percentage of flow up the graft during one cardiac cycle. The higher the BF the higher the competitive flow with a BF higher than 3% considered abnormal. A less commonly used TTFM parameter is the fast Fourier transform (FFT) ratio which is based on the analysis of the blood flow curve on TTFM. The major benefit of using FFT to predict patency is that it is relatively “resistant” to competitive flow compared to the other TTFM parameters, both in a multiscale modal [18] and in clinical data [19]. Of note, TTFM values are affected by blood pressure during the measurement – there is a direct correlation between blood pressure and flow.

Clearly, TTFM is not a perfect test. One of the main “Achilles heels” of TTFM is both false positive and false negative results. False negative results are usually the result of an issue with the toe of the anastomosis and poor flow to the distal part of the target artery but good flow proximal to the anastomosis [3]. Occlusion of the native coronary artery or combined use of TTFM and HFUS will usually help in discovering this. A false positive result (i.e., good graft with poor TTFM parameters), is often the result of competitive flow. The combined use of native coronary artery occlusion, HFUS, measurement of TTFM parameters with the cross clamp on (easily done with in situ IMA grafts), and most importantly surgeon experience, can all help reduce the amount of unnecessary graft revisions [20].

## Differences in flow parameters between arterial and venous grafts

Since there are different patency rates in different conduits, one might expect that there will be different flow parameters in arterial and venous conduits. Balacumaraswami et al. [21] measured MGF in 266 grafts in 100 patients undergoing CABG (80% of the patients underwent off pump surgery: OPCABG). They recorded similar flows in the IMA and the radial artery (RA, 29 and 31 mL/min, respectively). Both had significantly lower MGF than saphenous vein grafts (SVG) which was 47 mL/min. Flows were generally lower in patients undergoing OPCABG but the flow differences between arterial and venous conduits remained. This held true even when comparing only the RA and the SVG grafted to the obtuse marginal (OM) and the posterior descending artery (PDA). The same group later studied the flow profiles in arterial and venous conduits, but this time focusing on the left coronary territory [22]. In 506 grafts to the left system (divided equally between on and off pump CABG, 306 arterial grafts vs. 170 venous grafts), there was no statistically significant difference in MGF between arterial and venous grafts (43.6 ± 31.4 mL/min vs. 48.2 ± 33.6 mL/min, respectively). PI was also similar between the conduits though venous grafts had a higher PI in the propensity adjusted multivariable analysis. The DF was higher in arterial grafts when compared to venous grafts (71.0 ± 7.9% vs. 63.7 ± 11.1%, respectively). The percentage of BF was also higher in arterial grafts vs. SVG in the overall (2.3 ± 3.2% vs. 1.7 ± 3.2%, respectively). When comparing flow parameters in different surgical techniques, arterial conduits had a higher MGF and lower DF in on-pump CABG. Silva et al. [23] conducted a meta-analysis on flow profiles in CABG. They found 25 studies (19 observational and 6 randomized trials) with 4443 patients that were mainly focused on MGF. Of these, 15 studies focused on comparing flows in arterial vs. venous conduits. MGF was lower in arterial conduits when compared to venous conduits (standardized mean difference: −0.28; 95% CI [− 0.34; −0.22]; *P* < .001). It should be noted that in the studies used to examine MGF the *I*^*2*^ was 96%, suggesting considerable heterogeneity in the included studies.

## Changes in TTFM parameters after protamine

Several studies examined TTFM parameters before and after protamine infusion. In a retrospective analysis of the REQUEST database [7], Leviner et al. [24] showed that MGF and PI were unchanged in arterial grafts between the two measurements while there was a slight increase in MGF and slight decrease in PI in venous grafts after protamine administration. Dallan et al. [25], reporting the results of TTFM measurements in 575 patients with 1686 grafts (59.6% SVG) showed no statistically significant difference in either MGF or PI in arterial grafts after protamine infusion. They also saw no difference in post protamine MGF in venous grafts but a slight increase in PI.

## Skeletonized vs. pedicled IMA

There are two common harvesting techniques for the IMA – skeletonized and pedicled. While a pedicled graft is quicker and less technically demanding to harvest, there are concerns regarding an elevated risk of wound infection, especially when using BIMA. Two studies measured MGF in skeletonized vs. pedicled LIMA. The smaller study [26] (10 patients in each group) showed no significant difference between the two harvesting techniques while the larger study [27] (100 patients in each group) showed higher MGF and lower PI in the skeletonized group. Of interest, both groups showed considerable improvement in LIMA flow from initial preparation, after use of a vasodilator, and upon completion of the distal anastomosis.

## Comparison of graft revision in arterial compared to venous grafts

Another retrospective review of the REQUEST database [28] looked at differences in graft revision rates between arterial and venous grafts. There were 1105 (41.3%) venous grafts and 1570 (58.7%) arterial grafts in the database. There were more revisions in arterial vs. venous grafts (4.7% vs. 2.4%) with the most common type of revision being a primary anastomotic revision (defined as “Revision of the proximal or distal anastomosis due to a primary technical problem with the anastomosis itself and not due to a problem with the conduit” in the original manuscript [7]). The authors attribute this to the higher technical difficulty of arterial grafts and to the arterial conduits’ higher susceptibility to competitive flow and vasospasm which make TTFM interpretation difficult.

## Fractional flow reserve (FFR) in arterial grafts

Whereas there is ample evidence to support the use of FFR in assessing the physiologic significance of coronary stenosis and guiding PCI [29, 30], it’s use in guiding CABG has been less clear. While some studies have shown a good correlation between FFR and graft patency [31] and subsequently better clinical outcomes [32], this was not the case in the FARGO trial [33], which showed similar clinical outcomes and graft failures in FFR guided CABG compared to angiographically guided CABG. Relevant to our discussion, 67% of grafts in FARGO were venous, which are less susceptible to competitive flow. The IMPAG trial [34], in contrast, which measured the impact of preoperative FFR on graft function, focused only on arterial grafts and showed a very strong correlation between preoperative FFR and graft patency, with a cut-off of 0.78.

## Real world results with the use of TTFM in patients with total arterial revascularization

The impact of TTFM use in patients undergoing TAG was assessed in several large databases. Kieser et al. [35] prospectively enrolled 336 patients with 1000 grafts (99% arterial) and measured TTFM parameters in 990 of the grafts. All technical components of MAG and TAG were addressed with special attention to sequential and composite grafts (i.e., Y and T grafting), with the major take home message being that a PI > 5 was the strongest predictor of clinically significant graft dysfunction and showed a good correlation with post-operative adverse events. Laali and colleagues [36] retrospectively reviewed 910 patients who underwent TAG in their institution. In 430 of these patients TTFM was used (based on surgeon preference). TTFM use was associated with slightly longer operative times (cardiopulmonary bypass time of 76.0 min [62.0; 91.2] vs. 79.0 min [65.0; 94.0]). Six patients (1.4%) underwent graft revision in the TTFM group compared to no patients in the no TTFM group. The authors emphasize that patients undergoing graft revision had no other signs of graft dysfunction apart from abnormal TTFM parameters. This revision rate translated to a significantly lower rate of MACE (3.3% in the TTFM group vs. 6.9% in the no TTFM group). The authors also emphasize that use of TTFM in patients with TAG makes measurement on the arrested heart easier (since at least one of the grafts is in situ and can be allowed to flow while still with the cross clamp applied) and allows for an initial test of the graft at an early stage such that the decision to revise the graft might be less costly. Of course, grafts need to be reassessed with the heart full to make sure that the length and lye of the grafts does not compromise graft function.

## Clinical scenarios with the use of TTFM

To assist the readers in the early use of TTFM and to “shorten” the learning curve, we offer some common clinical scenarios with TTFM use in arterial grafts:

1. What should a surgeon do if LIMA to LAD flow is compromised by competitive flow from a venous conduit grafted to a diagonal branch? This scenario may be best avoided by grafting the diagonal with an arterial conduit as well, either a composite or sequential IMA or radial artery, thus avoiding the inherent initial difference in flow between arterial and venous grafts.
2. What should a surgeon do in the rare case of coronary - coronary steel seen with composite ITA grafting, where the lesion of one of the grafted coronaries turns out to be not as severe as predicted? Again, avoiding this in the first place by not constructing composite grafts when the stenosis is not severe is the best option. If already done, and the coronary-to-coronary steel is compromising the flow to the LAD than transecting the graft not supplying the LAD and using it as a free graft may be considered.

## Expert consensus recommendations for standardization of TTFM

Given the variations in reported TTFM measurements, as discussed above, an expert consensus statement, by Gaudino et al. [4], has proposed standardization to improve performance and interpretation of TTFM. In this document, 19 experts from around the world formulated 10 expert statements based on a 3-step Deplhi method (this method was used since large heterogeneity within individual studies made a meta-analysis inappropriate). Key recommendations included that: TTFM should be used in every CABG case (and not only in select cases since the surgeon needs to be thoroughly familiar with the technology in all situations from simple to complex scenarios); the TTFM probe should be positioned at the mid or distal part of the graft; TTFM measurements should be performed just before or after protamine administration and just before chest closure; questionable measurements should be repeated with a mean arterial pressure over 80 mmHg; a mean graft flow (MGF) < 15–20 mm/min and a pulsatility index (PI) > 3-3.5 should prompt further evaluation of a graft; and finally, the decision to revise a graft should not be based solely on TTFM readings but in the context of the full clinical scenario. For example, while listing the most common reasons for abnormal TTFM readings, the experts cite “poor distal runoff” as the second most common cause, emphasizing the need to include the size and runoff of the target vessel as a factor when using TTFM. Of note, the authors also remined us that while there is an added cost to the use of TTFM and there is no published cost effectiveness analysis of TTFM use, “…its cost/benefit ratio seems largely favorable, in view of the potential clinical consequences of graft dysfunction.”

## Conclusion

Increasing the use of arterial conduits should be a continuous goal to improve coronary surgery. Coupling this with the use TTFM in a methodical manner can potentially allow better short- and long-term outcomes in these technically demanding operations.

## Data Availability

Not applicable.
