# Correction: Transit time flow measurement in arterial grafts

**DOI:** 10.1186/s13019-024-02785-w

**Published:** 2024-05-10

**Authors:** Dror B. Leviner, John D. Puskas, David P. Taggart

**Affiliations:** 1 Department of Cardiac Surgery, Carmel Medical Center, Haifa, Israel; 2 The Ruth & Baruch Rappaport Faculty of Medicine, Technion-Israel Institute of Technology, Haifa, Israel; 3 Devision of Cardiothoracic Surgery, Emory University School of Medicine, Atlanta, GA USA; 4 Department of Cardiac Surgery, John Radcliffe Hospital, University of Oxford, Oxford, UK

**Journal of Cardiothoracic Surgery (2024) 19:224**

10.1186/s13019-024-02670-6

Following publication of the original article [1], the title was incorrectly given as ‘Transient time flow measurement in arterial grafts’ but should have been ‘Transit time flow measurement in arterial grafts’.

The original article has been corrected.
